# Diversity of Plasmids Encoding Virulence and Resistance Functions in *Salmonella enterica* subsp. *enterica* Serovar Typhimurium Monophasic Variant 4,[5],12:i:- Strains Circulating in Europe

**DOI:** 10.1371/journal.pone.0089635

**Published:** 2014-02-26

**Authors:** Patricia García, Katie L. Hopkins, Vanesa García, Janine Beutlich, M. Carmen Mendoza, John Threlfall, Dik Mevius, Reiner Helmuth, M. Rosario Rodicio, Beatriz Guerra, on behalf of the Med-Vet-Net WP21 Project Group

**Affiliations:** 1 Department of Functional Biology, Area of Microbiology, University of Oviedo, Oviedo, Asturias, Spain; 2 Antimicrobial Resistance and Healthcare Associated Infections Reference Unit, Public Health England (PHE), London, United Kingdom; 3 Department of Biological Safety, Federal Institute for Risk Assessment (BfR), Berlin, Germany; 4 Department of Bacteriology and TSEs, Central Veterinary Institute (CVI), Lelystad, The Netherlands; Indian Institute of Science, India

## Abstract

Plasmids encoding resistance and virulence properties in multidrug resistant (MDR) *Salmonella enterica* (*S*.) serovar Typhimurium monophasic variant 4,[5],12:i:- isolates recovered from pigs and humans (2006-2008) in Europe were characterised. The isolates were selected based on the detection by PCR-amplification of *S*. Typhimurium virulence plasmid pSLT genes and were analysed by multi-locus sequence typing (MLST). The resistance genes present in the isolates and the association of these genes with integrons, transposons and insertion sequences were characterised by PCR-sequencing, and their plasmid location was determined by alkaline lysis and by S1-nuclease pulsed-field gel electrophoresis (PFGE) Southern-blot hybridisation. Plasmids were further analysed by replicon typing, plasmid MLST and conjugation experiments. The 10 *S*. 4,[5],12,i:- selected isolates belonged to ST19. Each isolate carried a large plasmid in which MDR with pSLT-associated virulence genes were located. After analysis, eight different plasmids of three incompatibility groups (IncA/C, IncR and IncF) were detected. Two IncA/C plasmids represented novel variants within the plasmid family of the *S*. 4,[5],12:i:- Spanish clone, and carried an empty class 1 integron with a conventional *qacE*Δ*1*-*sul1* 3′ conserved segment or an In-*sul3* type III with *estX-psp-aadA2-cmlA1-aadA1-qacH* variable region linked to *tnpA440-sul3*, part of Tn*2*, Tn*21* and Tn*1721* transposons, and IS*CR2*. Four newly described IncR plasmids contained the resistance genes within In-*sul3* type I (*dfrA12-orfF-aadA2-cmlA1-aadA1-qacH*/*tnpA440*-*sul3*) and part of Tn*10* [*tet*(B)]. Two pSLT-derivatives with FIIs-ST1+FIB-ST17 replicons carried *cmlA1*-[*aadA1-aadA2*]-*sul3-dfrA12* and *bla*_TEM-1_ genes linked to an In-*sul3* type I integron and to Tn*2*, respectively. In conclusion, three emerging European clones of *S*. 4,[5],12:i:- harboured MDR plasmids encoding additional virulence functions that could contribute significantly to their evolutionary success.

## Introduction

The emergence of multiple clones of *Salmonella enterica* (*S*.) serovar Typhimurium-like strains lacking expression of the second flagellar antigen (*S*. 4,[5],12:i:- throughout the manuscript) has been reported worldwide [1]. Since the mid-1990s in Europe, *S*. 4,[5],12:i:- isolates have been increasingly implicated in human disease, and pig and pork products play an important role as a source of infection [1], [2]. Given the increase in their prevalence, these isolates represent a public health hazard and have been included in the European *Salmonella* control systems (http://eur-lex.europa.eu Accessed 1 January 2014). In addition, the two major clones circulating in Europe (labelled as Spanish and European clones) show multidrug resistance (MDR) to four or more unrelated classes of antimicrobials, making continued surveillance of their emergence and spread particularly important. Isolates belonging to the European clone exhibit resistance to ampicillin, streptomycin, sulphonamides and tetracycline (tetraR-type AMP-STR-SUL-TET) due to the presence of a resistance region on the bacterial chromosome, and lack the typical *S*. Typhimurium virulence plasmid (94 kb, pSLT) [1]–[4]. In contrast, the isolates belonging to the Spanish clone show a MDR phenotype with additional resistance to chloramphenicol, gentamicin and trimethoprim (AMP-CHL-GEN-STR/SPE-SUL-TET-TMP or variants therein) mediated by large non-conjugative plasmids (IncA/C or IncA/C+IncN plasmids of the pUO-STmRV1-like group) carrying the *spv* locus of the *Salmonella* virulence plasmids [1], [5], [6]. In order to investigate the presence and diversity of plasmids encoding resistance and virulence properties, which could give a selective advantage to emerging clones of *S*. 4,[5],12:i:- in Europe, isolates collected from different sources and countries within the European Union Network of Excellence (NoE) Med-Vet-Net were analysed.

## Materials and Methods

### Bacterial isolates and properties

The isolates included in this work belong to the *S*. 4,[5],12:i:- European NoE Med-Vet-Net (www.medvetnet.org) WP21 collection. This collection [4], included 116 *Salmonella* monophasic isolates recovered from human, pig and pork products (2006–2008) provided by eight participating laboratories from six countries (Public Health England, and the Animal Health and Veterinary Laboratories Agency, UK; the Agence Française de Sécurité Sanitaire des Aliments, France, the Federal Institute for Risk Assessment, Germany, the Istituto Superiore di Sanità, Italy; the National Institute of Public Health in Warsaw, Poland; the Health Surveillance Centre (VISAVET), University Complutense in Madrid, Spain and the Central Veterinary Institute of Wageningen, the Netherlands). Data on features like phage type, antimicrobial resistance pattern, XbaI-PFGE pattern and multiple-locus variable-number tandem repeat analysis (MLVA) profile of the isolates, were previously published [4]. Since the aim of the present study was the detection and further characterization of plasmids containing resistance and virulence genes, we followed two criteria for selection within this collection: MDR phenotype different than the tetraR-type of the European clone (characteristically lacking the virulence pSLT plasmid) together with the detection of the *spvC* gene (gene widely used as a molecular marker for the detection of pSLT or pSLT-derivatives [7]). Only *spvC*-positive isolates (10 isolates) were suspected to contain a hybrid plasmid and were further analyzed. Multilocus sequence typing (MLST) was performed in the present study according to the website database recommendations (http://mlst.ucc.ie/mlst/dbs/Senterica).

### Antimicrobial susceptibility testing, detection of resistance genes and characterisation of their genetic environments

The isolates were tested by broth microdilution for their antimicrobial susceptibility against a panel of 14 antimicrobials (ampicillin, cefotaxime, ceftazidime, chloramphenicol, ciprofloxacin, colistin, florfenicol, gentamicin, kanamycin, nalidixic acid, streptomycin, sulfamethoxazole, tetracycline, and trimethoprim) as previously described [8]. The results were analysed following the cut-offs set by EUCAST (www.eucast.org). Genes responsible for resistance to ampicillin [*bla*_PSE-1_, *bla*_OXA-1-like_, *bla*_TEM-1-like_], chloramphenicol [*catA1*, *cmlA1*, *floR*], aminoglycosides [*aac(3)-IV*, *aadA1-like*, *aadA2*, *strA*, *strB*], sulphonamides [*sul1*, *sul2*, *sul3*], tetracycline [*tet*(A), *tet*(B)] and trimethoprim [*dfrA1*-like, *dfrA12*] were studied by multiplex and simplex PCR-amplification [8]. The genetic environments were characterized by overlapping PCR-amplification and sequencing (Table S2 and Figure S1).

### Plasmid content and characterisation of ‘resistance-virulence’ plasmids

The plasmids were classified by PCR-based replicon typing including IncU and IncR replicons [9], [10], and if appropriate, by plasmid MLST (pMLST, http://pubmlst.org/plasmid/). The conserved scaffold of IncA/C plasmids was screened by PCR as reported previously [11]. pSLT-genes encoding virulence functions other than *spvC* (*rck*, *mig5* and *srgB*) were PCR tested. The plasmid content of the *S*. 4,[5],12:i:- isolates (and transconjugants if applicable) was determined by alkaline lysis and S1-nuclease-PFGE. The profiles obtained were subsequently analysed by Southern-blot hybridisation [6] using specific probes for *spvC*, 12 resistance genes [*bla*_TEM-1-like_, *cmlA1*, *aac(3)-IV*, *aadA1*-like, *aadA2*, *strA*, *sul1*, *sul2*, *sul3*, *tet*(A), *tet*(B), *dfrA12*], five transposon/IS*CR* markers [*merA*-Tn*21*, *tnpR*-Tn*1721*, *tnpR-bla*-Tn*2*, *tetR*-Tn*10* and *rcr2*-IS*CR2*], and four Inc replicons [A/C, R, FIIs and FIB]. Plasmids containing *spvC* plus resistance genes were labelled as pMVN-STmRV (1 to 4) and pMVN-STmVR (1 to 2).

### Mating experiments

The horizontal transmission capabilities of the plasmids were investigated by conjugation experiments. For this analysis six strains (two per VR- or RV- plasmid incompatibility group detected) were chosen. These strains carried the following plasmids: pMVN-STmRV1 and pMVN-STmRV2 of IncA/C (160 kb); pMVN-STmRV3 (100 kb) and pMVN-STmRV6 (160 kb) of IncR and pMVN-STmVR1 (130 kb) and pMVN-STmVR2 (160 kb) of IncF. Mating assays were performed in liquid broth (30°C and 37°C) for 20 h, using *S*. 4,[5],12:i:- strains as donors and rifampicin-resistant *E. coli* K-12 J53 as recipient. Transconjugants were selected on Eosin-Methylene Blue agar plates containing rifampicin (50 mg/L) plus either chloramphenicol (30 mg/L), sulphonamides (300 mg/L), tetracycline (30 mg/L) and/or trimethoprim (10 mg/L).

## Results and Discussion

In *Salmonella enterica*, resistance plasmids containing the *spv* region appear to have evolved either by acquisition of virulence genes by resistance plasmids of different incompatibility groups (RV) or through the integration of resistance islands into serovar-specific virulence plasmids (VR) [12]. In this study, both types were detected among 10 *S*. 4,[5],12:i:- isolates recovered in the United Kingdom, Spain and Italy. The isolates were epidemiologically unrelated [4] excepting two isolates from Italy which, as determined in this work, could be considered as one strain (Table 1 and S1). All isolates were assigned by MLST to ST19, which constitutes the main ST found in diphasic *S*. Typhimurium (http://mlst.ucc.ie/mlst/dbs/Senterica) and in other monophasic isolates with plasmid-encoded MDR [13]. In contrast, monophasic isolates assigned to the European clone, characterised by the chromosomally-encoded AMP-STR-SUL-TET phenotype, usually belong to ST34 and lack the serovar-specific virulence plasmid [1]–[4]. Of note, MLST analysis suggests that the Spanish and other plasmid-harbouring clones, such as those detected in this study, originated independently from one or more *S*. Typhimurium ancestor(s) distinct from that of the European clone.

**Table 1:** Resistance plasmids with pSLT genes identified in this study and properties of the host isolates.

						Isolates (isolation	
pMVN-	Size	Inc-	Resistotype conferred to the host^b^	5′CS/gene cassettes^c^/3′CS^d^	pSLT-	year)^g^/Country^h^/	Other R-
STm^a^	(∼kb)	group	(phenotype/genotype)	Tn-*like* and IS*CR* e	genes^f^	Source^i^	plasmids^k^
			AMP, GEN, SUL, TET/	-/none/*qacEΔ1*-*sul1*			
RV1	160	A/C	*bla*_TEM-1_, *aac(3)-IV*, [*sul1, sul2*], *tet*(A)	Tn*2*-, Tn*1721*-, Tn*21-like*, IS*CR2*	*spvC, mig5*	H07-0207(07)/UK/Hu	None
				*intI1*/none/*qacEΔ1*-*sul1*			
			AMP, CHL, GEN, [STR, SPE], SUL, TET*/*	*intI1*/type III/*tnpA440-sul3*			100 kb- FIB(ST20)
RV2	160	A/C	*bla*_TEM-1_, *cmlA1*, *aac(3)-IV*, [*aadA1, aadA2*], [*sul1, sul2, sul3*], *tet*(A)	Tn*2*-, Tn*1721*-, Tn*21-like*, IS*CR2*	*spvC, mig5*	RL0-0511(06)/E/Sw	*bla*_TEM-1_
			CHL, [STR, SPE], SUL, TET, TMP/	*intI1*/type I/*tnpA440-sul3*			
RV3	100	R	*cmlA1*-[*aadA1*-*aadA2*]-*sul3*-*tet*(B)-*dfrA12*	Tn*10-like*	*spvC*	RL0-0527(06)/I/Hu	None
			CHL, [STR, SPE], SUL, TET, TMP/	*intI1*/type I/*tnpA440-sul3*			
RV4	120	R	*cmlA1*, [*aadA1, aadA2*], *sul3*, *tet*(B), *dfrA12*	Tn*10-like*	*spvC*	RL0-0532(06)/I/Hu	None
			CHL, [STR, SPE], SUL, TET, TMP/	*intI1*/type I/*tnpA440-sul3*			
RV5	130	R	*cmlA1*, [*aadA1, aadA2*], *sul3*, *tet*(B), *dfrA12*	*Tn10-like*	*spvC, mig5*	RL0-0513(06)/E/Sw	None
			CHL, [STR, SPE], SUL, TET, TMP/	*intI1*/type I/*tnpA440-sul3*		RL0-0530(06)/I/Hu	None
RV6	160	R	*cmlA1*, [*aadA1, aadA2*], *sul3, tet*(B), *dfrA12*	Tn*10-like*	*spvC, mig5*	RL0-0535(07)/I/Hu^j^	
						RL0-0536(07)/I/Hu^j^	None
		FIIs-ST1	AMP, CHL, [STR, SPE], SUL, TMP/	*intI1*/type I/*tnpA440-sul3*	*spvC*, *rcK*,		∼20 kb-nd-
VR1	130	+FIB-ST17	*bla*_TEM-1_, *cmlA1*, [*aadA1, aadA2*], *sul3, dfrA12*	Tn*2-like*	*mig5*, *srgB*	RL0-0490(07)/UK/Sw	*strA*+*sul2*+*tet*(A)
		FIIs-ST1	AMP, CHL, [STR, SPE], SUL, TMP/	*intI1*/type I/*tnpA440-sul3*	*spvC*, *rcK*,		∼20 kb-nd-
VR2	150	+FIB-ST17	*bla*_TEM-1_, *cmlA1*, [*aadA1, aadA2*], *sul3, dfrA12*	Tn*2-like*	*mig5*, *srgB*	RL0-0500(07)/UK/Sw	*strA*+*sul2*+*tet*(A)

a pMVN-STmRV/VR, plasmid Med-Vet-Net *Salmonella* Typhimurium monophasic resistance-virulence/virulence-resistance. The horizontal transmission capabilities were investigated by conjugation experiments for the following plasmids: RV1, RV2, RV3, RV6, VR1 and VR2; as described in Materials and Methods.

b All resistance genes were demonstrated as plasmid located by Southern blot hybridization on plasmid profiles. Antimicrobial abbreviations: AMP, ampicillin; CHL, chloramphenicol; GEN, gentamicin; STR, streptomycin; SPE, spectinomycin; SUL, sulphonamides; TET, tetracycline and TMP, trimethoprim.

c Gene cassette organization of unusual *sul3*-integrons are: In-*sul3*-type I, *dfrA12-orfF-aadA2-cmlA1-aadA1-qacH* and In-*sul3*-type III, *estX-psp-aadA2-cmlA1-aadA1-qacH*.

d Other class 1 integron related sequences tested were *intI1* (5′CS), and *qacE*Δ*1*, *tnpA440, sul1* and *sul3* (3′CS).

e Transposon and insertion sequence common region gene markers tested by Southern blot hybridization were: *tnpR* (Tn*1721*), *tnpR-bla* (Tn*2*), *merA* (Tn*21*), *tetR* (Tn*10*) and *rcr2* (IS*CR2*). Other genes located on the same mobile genetic element were tested by simple and overlapping PCR amplifications as indicated in Table S2, Table S3 and Figure S1.

f Virulence genes tested were: *spvC*, *rck*, *mig5* and *srgB* characteristic of pSLT virulence plasmid. The *spvC* gene was mapped on S1- and alkaline lysis-plasmid profiles.

g The sender code and properties already published for each strain is shown in Table S1.

h Countries abbreviations (sender Laboratory): UK, the United Kingdom (PHE, Public Health England [formerly HPA, Health Protection Agency] and AHVLA, Animal Health and Veterinary Laboratories Agency); E, Spain (VISAVET-UCM, Centro de Vigilancia Sanitaria Veterinaria-Universidad Complutense de Madrid) and I, Italy (ISS, Istituto Superiore di Sanità).

i Source abbreviations: Hu, human; Sw, swine.

j Both isolates have shown identical properties, hence considered as one strain.

k nd, Inc group not determined. Other small (<30 kb) co-resident plasmid but with no resistance genes were detected by alkaline lysis (data not shown).

In this study, both resistance-virulence and virulence-resistance plasmids, with sizes ranging from ∼100 to 160 kb, were identified in *S*. 4,[5],12:i:- (Table 1). No isolate carried a 94 kb plasmid consistent with pSLT. Each isolate was found to contain a RV or VR plasmid belonging to incompatibility groups IncA/C, IncR or IncF, with or without other co-resident plasmids.

### IncA/C plasmids

**Figure 1:**
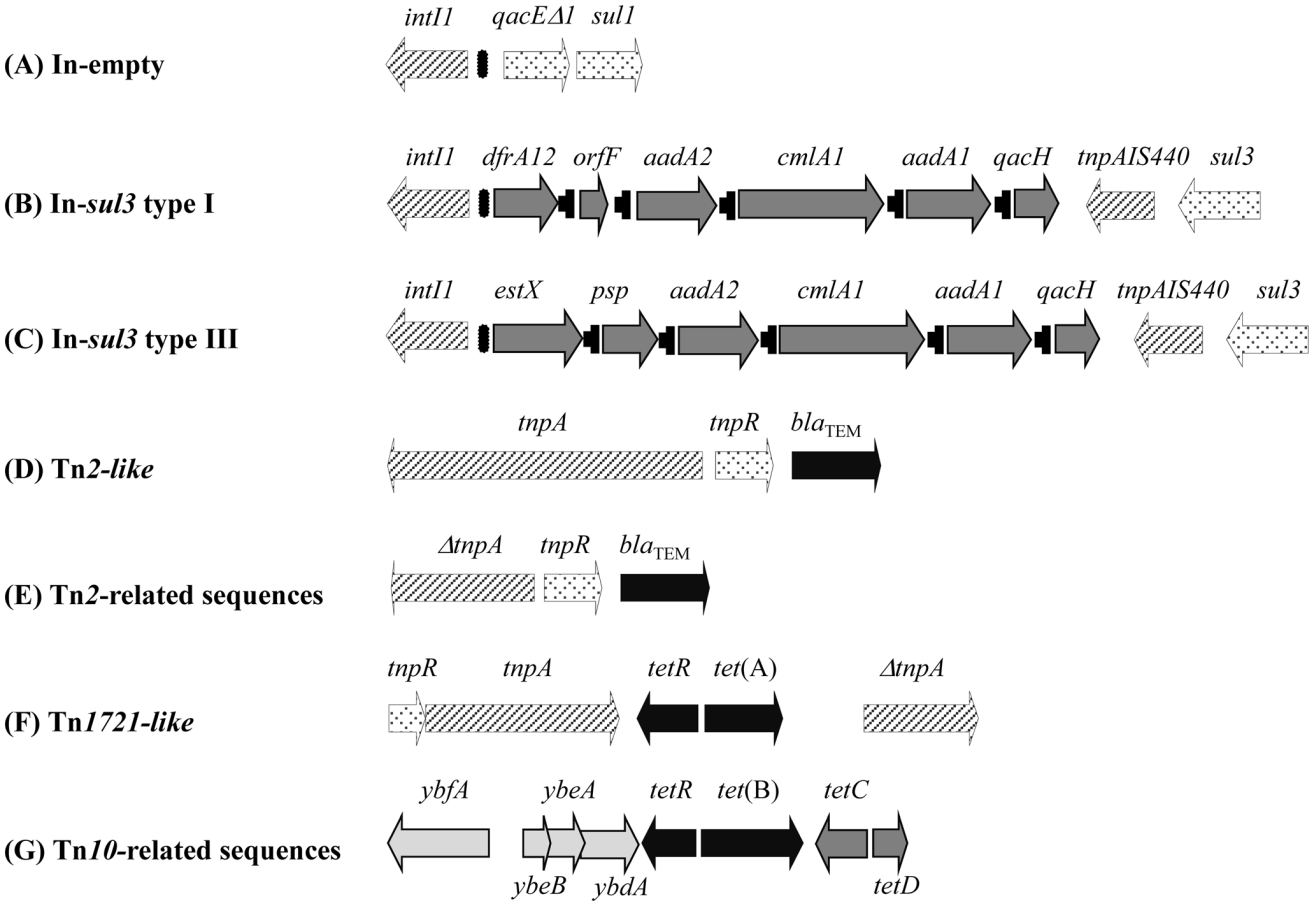
Schematic overview of the integrons (A to C) and transposons (D to G) detected in this study. The information of which plasmid carried each structure is shown in Table 1. An overview of PCR mapping strategy designed to establish the structure and primers used are shown in Figure S1 and Table S2, respectively. Please note that the scale is not the same for all schemes.

Two 160 kb IncA/C plasmids, named pMVN-STmRV1 (found in a human isolated from the UK) and pMVN-STmRV2 (from a swine isolate from Spain), were found. According to their sizes, resistance genes and mobile genetic elements carried, they represent two new variants of the pUO-STmRV1-like group typically found in the Spanish *S*. 4,[5],12:i:-clone [6] (Table 1, Figure 1, Table S3). These two plasmids were the only ones to carry the *aac(3)-IV* gene conferring gentamicin and tobramycin resistance, a defective Tn*1721* [*tet*(A)] transposon (Figure 1F), Tn*21*-related sequences (*tnpM*, *tnpR* and *merA*, but negative for the transposase gene), and IS*CR2* linked to *sul2*. The sequence of the A/C replicon amplicon (437 bp, accession numbers HF968758 and HF968759) of both plasmids was identical to the corresponding fragment of the *repA* gene of pUO-STmRV1 (accession number HF968757) [6]. This A/C subtype differs in 22 and five nucleotides from the A/C_1_ and A/C_2_ replicons [14] affecting the encoded RepA proteins, with one and two amino acid variations, respectively [6]. Similar to pUO-STmRV1, pMVN-STmRV1 and pMVN-STmRV2 lacked part of the IncA/C conserved backbone including several genes of the *tra* region (PCR profiles according to Welch *et al*. [11]: pUO-STmRV1 [1, 2, 3, 4, −, −, −, −, −, 10, 11, 12], pMVN-STmRV1 [1, 2, 3, 4, −, −, −, −, 9, 10, 11, 12] and pMVN-STmRV2 [1, 2, 3, −, −, −, −, −, 9, 10, 11, −]). Consequently, they were not self-transferable, though pMVN-STmRV2 generated a larger co-integrated plasmid after the conjugation process (as demonstrated by hybridisation of the plasmid DNA; data not shown), suggesting that it could be mobilized by the co-resident IncFIB-ST20 R-plasmid (*bla*_TEM-1_) of ∼100 kb. In agreement with the pUO-STmRV1 group, they were also negative for the *rck* and *srgB* genes of pSLT. In summary, the two new plasmid variants described here support the presence of *S*. 4,[5],12:i:- isolates of the Spanish clone in the United Kingdom. Moreover, evidence of their occurrence in Portugal and France has already been reported [15], [16].

### IncR plasmids

Six other isolates (five strains) collected in Italy (human) and Spain (swine) contained four distinct plasmids with the IncR replicon (pMVN-STmRV3-RV6). These plasmids shared a common resistance pattern but differed in size (100 to 160 kb) and pSLT-related genes (Table 1). All resistance genes harboured by them, with the exception of *tet*(B), formed part of a *sul3*-type I integron (*intI1*/*dfrA12-orfF-aadA2-cmlA1-aadA1-qacH/tnpA440-sul3*). The *tet*(B) gene was found associated with Tn*10*-related sequences (Figure 1B,G and Table S3). Like other reported IncR plasmids, they appear to be non-conjugative and, together with resistance functions, the acquisition of virulence genes from pSLT indicates their ability to capture additional genetic information that could bring an advantage to the host. As shown by the present study, IncR plasmids represent a new genetic platform that will allow co-selection of both virulence and resistance functions. Since the definition of the IncR family in 2009 [10], resistance plasmids belonging to this family and linked to quinolone and extended-spectrum β-lactam resistance, have been increasingly reported in enteric pathogens. However, to our knowledge this is the first report describing IncR plasmids in *S*. 4,[5],12:i:- isolates, and in fact, IncR was the most frequent replicon found in our collection.

### IncF pSLT-derivatives

The two remaining isolates found in the United Kingdom (swine), carried plasmids with both IncFIIs-ST1 and IncFIB-ST17 replicons. These plasmids (pMVN-STmVR1 and pMVN-STmVR2), of 130 and 150 kb respectively, contained all pSLT genes tested (*spvC*, *rcK*, *mig5* and *srgB*) and could have been derived from pSLT through acquisition of multiple resistance genes (Table 1). In both, *bla*_TEM-1_ was linked to an apparently complete copy of Tn*2*, while the remaining genes were associated with a *sul3*-type I integron (*intI1*/*dfrA12-orfF-aadA2-cmlA1-aadA1-qacH/tnpA440-sul3*) (Figure 1B,D and Table S3). Conjugation assays demonstrated that only pMVN-STmVR2 was self-transferable under the conditions used. Both plasmids co-reside in the same cell with a 20 kb R-plasmid (carrying *strA*, *sul2* and *tet*(A) genes). Based on the resistance genes and associated genetic elements identified, pMVN-STmVR1 and pMVN-STmVR2 seem to be related to plasmids found in isolates of diphasic *S*. Typhimurium recovered from pigs in the UK in a parallel study [17], thus suggesting a genetic exchange between the two *S*. Typhimurium populations co-existing in the same environment. Resistance derivatives of the serovar-specific virulence plasmid of *S*. Typhimurium (pSLT) were found in diphasic *S*. Typhimurium isolates recovered from clinical and animal samples in different countries [12], [17], [18] but until now, not in isolates belonging to the monophasic variant.

## Conclusions

The public health risk associated with monophasic *S*. 4,[5],12:i:- in Europe is demonstrated by an increased isolation rate from food and human sources together with the MDR prevalence (http://eur-lex.europa.eu, Accessed 1 January 2014). However, so far, little is known about clones circulating in Europe which harbour resistance plasmids carrying the *spv* locus of pSLT. Emerging clones of *S*. 4,[5],12:i:- could have a selective advantage conferred not only by MDR properties but also by virulence factors linked together on the same plasmid. Accordingly, the role that these genetic platforms could play on the emergence and/or spread of this variant should be taken into consideration. In fact, within this study we have described and characterised eight new variants belonging to three Inc groups. Full sequencing methodologies will help to clarify the molecular events responsible for the construction of these and other complex mosaic plasmids that contribute significantly to the evolutionary success of certain pathogenic bacterial clones. Because several *S*. Typhimurium monophasic clonal lines have been increasingly associated with human infections, the continuous surveillance of their properties, distribution and spread is particularly important. In this context, the molecular data reported here underline the need of coordinated surveillance to provide additional information and shed light on the knowledge of circulating clones of *S*. 4,[5],12:i:- in Europe.

## Supporting Information

Figure S1 **Schematic overview of the PCR-mapping strategy designed to establish the structure of the integrons (A to E) and transposons (F to H).**
(DOC)

Table S1 **General properties and sender code of the isolates analyzed in this study.**
(DOC)

Table S2 **Primers used for simplex and overlapping PCRs performed to investigate integron-, transposon- and insertion sequence common regions-related sequences. Primers used for resistance and virulence genes are as indicated in footnote references.**
(PDF)

Table S3 **Overlapping PCRs designed to characterize integrons, transposons and IS** ***CR*** **elements.**
(PDF)

## References

[1] European Food Safety Authority. (2010) Scientific Opinion on monitoring and assessment of the public health risk of “*Salmonella* Typhimurium-like” strains. EFSA Journal. doi:10.2903/j.efsa.2010.1826.

[2] Hauser E, Tietze E, Helmuth R, Junker E, Blank K, et al (2010) Pork contaminated *Salmonella enterica* serovar 4,[5],12:i:-, an emerging health risk for humans. Appl Environ Microbiol 76: 4601–4610 10.1128/AEM.02991-09 20472721 PMC2901716

[3] Lucarelli C, Dionisi AM, Filetici E, Owczarek S, Luzzi I, et al (2012) Nucleotide sequence of the chromosomal region conferring multidrug resistance (R-type ASSuT) in *Salmonella* Typhimurium and monophasic *Salmonella* Typhimurium strains. J Antimicrob Chemother 67: 111–114 10.1093/jac/dkr391 21990047

[4] Hopkins KL, Kirchner M, Guerra B, Granier SA, Lucarelli C, et al. (2010) Multiresistant *Salmonella enterica* serovar 4,[5],12:i:- in Europe: a new pandemic strain?. Euro Surveill 15: pii = 19580. Available: http://www.eurosurveillance.org/ViewArticle.aspx?ArticleId=19580. Accessed 1 January. 20546690

[5] Guerra B, Soto SM, Arguelles JM, Mendoza MC (2001) Multidrug resistance is mediated by large plasmids carrying a class 1 integron in the emergent *Salmonella enterica* serotype [4,[5],12:i:-]. Antimicrob Agents Chemother 45: 1305–1308 10.1128/AAC.45.4.1305-1308.2001 11257054 PMC90463

[6] García P, Guerra B, Bances M, Mendoza MC, Rodicio MR (2011) IncA/C plasmids mediate antimicrobial resistance linked to virulence genes in the Spanish clone of the emerging *Salmonella enterica* serotype 4,[5],12:i:-. J Antimicrobial Chemother 66: 543–549 10.1093/jac/dkq481 21177672

[7] Herrero A, Mendoza MC, Threlfall EJ, Rodicio MR (2009) Detection of *Salmonella enterica* serovar Typhimurium with pUO-StVR2-like virulence-resistance hybrid plasmids in the United Kingdom. Eur J Clin Microbiol Infect Dis 28: 1087–1093 10.1007/s10096-009-0753-1 19444492

[8] Beutlich J, Jahn S, Malorny B, Hauser E, Hühn S, et al (2011) Antimicrobial resistance and virulence determinants in European *Salmonella* Genomic Island 1-positive *Salmonella enterica* isolates from different origins. Appl Environ Microbiol 77: 5655–5664 10.1128/AEM.00425-11 21705546 PMC3165277

[9] Carattoli A, Bertini A, Villa L, Falbo V, Hopkins KL, et al (2005) Identification of plasmids by PCR-based replicon typing. J Microbiol Methods 63: 219–228 10.1016/j.mimet.2005.03.018 15935499

[10] García-Fernández A, Fortini D, Veldman K, Mevius D, Carattoli A (2009) Characterization of plasmids harbouring *qnrS1*, *qnrB2* and *qnrB19* genes in *Salmonella* . J Antimicrob Chemother 63: 274–281 10.1093/jac/dkn470 19001452

[11] Welch TJ, Fricke WF, McDermott PF, White DG, Rosso M-L, et al (2007) Multiple antimicrobial resistance in plague: an emerging public health risk. PLoS One 2: e309 10.1371/journal.pone.0000309 17375195 PMC1819562

[12] Rodicio MR, Herrero A, Rodríguez, García P, Montero I, et al (2011) Acquisition of antimicrobial resistance determinants by virulence plasmids specific for non typhoid serovars of *Salmonella enterica* . Rev Med Microb 22: 55–65.

[13] García P, Malorny B, Hauser E, Mendoza MC, Rodicio MR (2013) Genetic types, gene repertoire and evolution of isolates of the *Salmonella* serovar 4,[5],12:i:- Spanish clone assigned to different phage types. J Clin Microbiol 51: 973–978 10.1128/JCM.02777-12 23325816 PMC3592032

[14] Carattoli A, Miriagou V, Bertini A, Loli A, Colinon C, et al (2006) Replicon typing of plasmids encoding resistance to newer beta-lactams. Emerg Infect Dis 12: 1145–1148 10.3201/eid1207.051555 16836838 PMC3291055

[15] Antunes P, Mourao J, Pestana N, Peixe L (2011) Leakage of emerging clinically relevant multidrug-resistant *Salmonella* clones from pig farms. J Antimicrob Chemother 66: 2028–2032 10.1093/jac/dkr228 21697179

[16] Bugarel M, Granier SA, Bonin E, Vignaud ML, Roussel S, et al (2012) Genetic diversity in monophasic (1,4,[5],12:i:- and 1,4,[5],12:-:1,2) and in non-motile (1,4,[5],12:-:-) variants of *Salmonella enteric*a *S*. Typhimurium. Food Research Inter 45: 1016–1024.

[17] Beutlich J, Rodicio MR, Mendoza MC, García P, Kirchner M, et al (2013) *Salmonella enterica* serovar Typhimurium virulence-resistance plasmids derived from the pSLT carrying nonconventional class 1 integrons with *dfrA12* gene in their variable region and *sul3* in the 3′ conserved segment. Microb Drug Resist 19: 437–445 10.1089/mdr.2012.0226 23808958

[18] Kingsley RA, Msefula CL, Thomson NR, Kariuki S, Holt KE, et al (2009) Epidemic multiple drug resistant *Salmonella* Typhimurium causing invasive disease in sub-Saharan Africa have a distinct genotype. Genome Res 19: 2279–2287 10.1101/gr.091017.109 19901036 PMC2792184

