# Supplementary material for: Diversity of Plasmids Encoding Virulence and Resistance Functions in Salmonella enterica subsp. enterica Serovar Typhimurium Monophasic Variant 4,[5],12:i:- Strains Circulating in Europe
Source: PLoS One. 2014 Feb 26;9(2):e89635. doi: 10.1371/journal.pone.0089635 (PMC3935914; doi:10.1371/journal.pone.0089635)
Supplement: Figure S1 — Schematic overview of the PCR-mapping strategy designed to establish the structure of the integrons (A to E) and transposons (F to H). (DOC) [file pone.0089635.s001.doc]

**
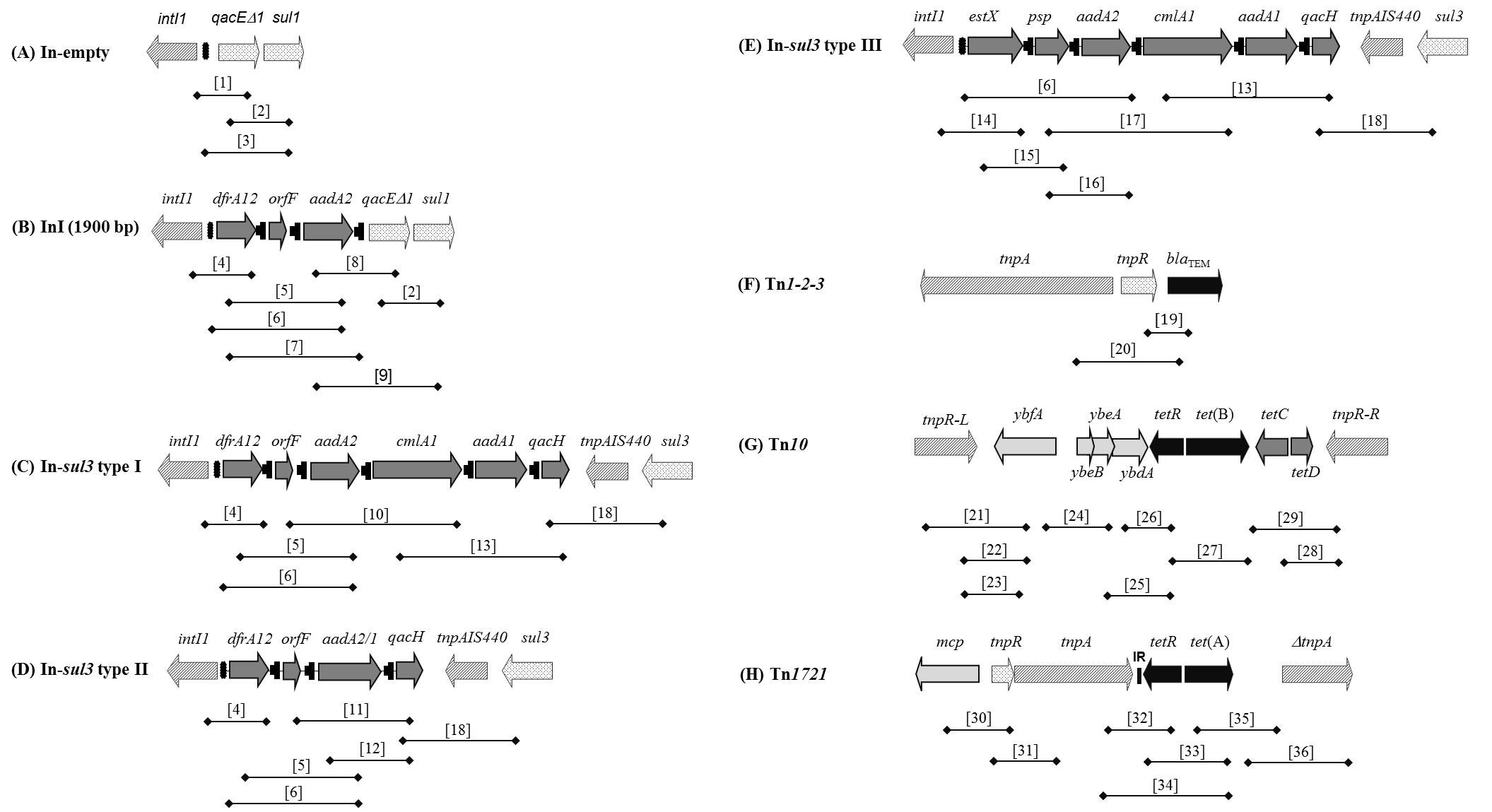
**

**Figure S1**. Schematic overview of the PCR-mapping strategy designed to establish the structure of the integrons (A to E) and transposons (F to H). Lines below each structure represent expected amplicons for the indicated PCR reaction (Table S2). Please note that the scale is not the same for all schemes, and that simplex PCR reactions are not included.
