# Supplementary material for: Diversity of Plasmids Encoding Virulence and Resistance Functions in Salmonella enterica subsp. enterica Serovar Typhimurium Monophasic Variant 4,[5],12:i:- Strains Circulating in Europe
Source: PLoS One. 2014 Feb 26;9(2):e89635. doi: 10.1371/journal.pone.0089635 (PMC3935914; doi:10.1371/journal.pone.0089635)
Supplement: Table S1 — General properties and sender code of the isolates analyzed in this study. (DOC) [file pone.0089635.s002.doc]

**Table S1.** **General properties and sender code isolates analyzed in this study.**

| **Isolate number** | **Isolation year** | **Source** | **Sender^a^** | **Sender strain code** | **Country^b^** | **Phage type^c^** | **PFGE pattern^c^** | **MLVA pattern^c^** |
| --- | --- | --- | --- | --- | --- | --- | --- | --- |
| H07-0207 | 2007 | Human | PHE | H07-0207 | UK | U302L | un-named | 3-15-13-NA-311 |
| RL0-0511 | 2006 | Swine | UCM | VE06/00692SK1 | E | U302 | 0246 | 3-12-18-NA-311 |
| RL0-0527 | 2006 | Human | ISS | 33/1/06 | I | 18 variant | 0083 | 13-13-NA-NA-211 |
| RL0-0532 | 2006 | Human | ISS | 80/9/06 | I | 18 variant | 0079 | 13-13-NA-NA-211 |
| RL0-0513 | 2006 | Swine | UCM | VE06/00829SK1 | E | 18 | un-named | 13-14-NA-9-211 |
| RL0-0530 | 2006 | Human | ISS | 33/12/06 | I | RDNC | un-named | 11-14-NA-8-211 |
| RL0-0535 | 2007 | Human | ISS | 35/7/07 | I | 120 | 0079 | 11-15-NA-8-211 |
| RL0-0536 | 2007 | Human | ISS | 35/8/07 | I | 120 | 0079 | 11-15-NA-8-211 |
| RL0-0490 | 2007 | Swine | AHVLA | S00040-07 | UK | 193 | 0218 | 5-19-9-10-211 |
| RL0-0500 | 2007 | Swine | AHVLA | S10023-07 | UK | 193 | un-named | 5-18-11-11-111 |

^a^Sender Laboratory abbreviations: PHE, Public Health England (formerly HPA, Health Protection Agency); VISAVET-UCM, Centro de Vigilancia Sanitaria Veterinaria-Universidad Complutense de Madrid; AHVLA, Animal Health and Veterinary Laboratories Agency; ISS, Istituto Superiore di Sanità.

^b^Country abbreviations: UK, United Kingdom; E, Spain; I, Italy.

^c^Phage type, PFGE and MLVA patterns as published previously [S1]. PFGE patterns, pulsed-field gel electrophoresis STYMXB. profiles named according to PulseNet Europe database. MLVA patterns, multi-locus variable tandem repeat analysis patterns follow the nomenclature developed by Larsson et al. [S2].

**S1.** Hopkins KL, Kirchner M, Guerra B, Granier SA, Lucarelli C, et al. (2010) Multiresistant *Salmonella enterica* serovar 4,[5],12:i:- in Europe: a new pandemic strain?. Euro Surveill 15: pii=19580. Available: <http://www.eurosurveillance.org/ViewArticle.aspx?ArticleId=19580>. Accessed 1 January 2014.

**S2**. Larsson JT, Torpdahl M, Petersen RF, Sorensen G, Lindstedt BA, et al. (2009) Development of a new nomenclature for *Salmonella* Typhimurium multilocus variable number of tandem repeats analysis (MLVA). Euro Surveill 2009; 14: pii=19174. Available: <http://www.eurosurveillance.org/ViewArticle.aspx?ArticleId=19174>. Accessed 1 January 2014.
