# Supplementary material for: Diversity of Plasmids Encoding Virulence and Resistance Functions in Salmonella enterica subsp. enterica Serovar Typhimurium Monophasic Variant 4,[5],12:i:- Strains Circulating in Europe
Source: PLoS One. 2014 Feb 26;9(2):e89635. doi: 10.1371/journal.pone.0089635 (PMC3935914; doi:10.1371/journal.pone.0089635)
Supplement: Table S2 — Primers used for simplex and overlapping PCRs performed to investigate integron-, transposon- and insertion sequence common regions-related sequences. Primers used for resistance and virulence genes are as indicated in footnote references. (PDF) [file pone.0089635.s003.pdf]

Table S2. Primers used for simplex and overlapping PCRs performed to investigate integron-, transposon- and insertion sequence common regions-related sequences. Primers used for resistance and virulence genes are as indicated in footnote references.

Genetic element	Target gene/region Function	Primer name	Sequence (5'-3')	Fragment Size (bp)	Reference
**Integrons**					
Class 1	Integrase type 1	intI1-F/R	GCCTTGCTGTTCTTCTAC/ GATGCCTGCTTGTTCTAC	558	S1
		intI-R2	GATGCCTGCTTGTTCTAC	[a]	This work
	Variable region of conventional integrons	5'CS/3'CS	GGCATCCAAGCAGCAAG/ AAGCAGACTTGACCTGA	variable	S1
	R-quaternary ammonium 3'CS conventional integrons	qacEΔ1-F/R	ATCGCAATAGTTGGCGAAGT/ CAAGCTTTTGCCCATGAAGC	250	S1
	Transposase of IS*440* 3'CS atypical integrons	tnpAIS440-F/R	GAAAGGAACGCCAGTTTACC/ CCATCGCCGAAATGTATGTC	323	S1
	R-quaternary ammonium gene cassette atypical integrons	qacH-F/R	TGTGCGCTGACCTTGGATAG/ TCCATGGCCAGAAACTAGAC	96	S1
	Hypothetical protein	orfF-F	AGCAAGCGCAAGCAGCTATC	[a]	S1
	Putative esterase gene cassette atypical integrons	estX-F/R	CGGCTTGCAGATGCAGATCC/ ATAATCCTCGGCCAGTCTTC	430	S1
	Phosphoserine phosphatase gene cassette atypical integrons	Psp-F/R	CTCTCGCACTCTTTGACTTC/ CAACCTCTTGCCACTGATAG	585	This work S1
Class 2	Integrase type 2	intI2-F/R	CACGGATATGCGACAAAAAGGT/ GTAGCAAACGAGTGACGAAATG	788	S2
**Transposons and insertion sequences common regions**					
Tn*21*	Transposase *tnpA*	tnpA-F/R	AGAAAGTTCGTCCTGGGCTG/ GGCCAAGGACAAGAACCTGT	317	S1
	Resolvase *tnpR*	tnpR-F/R	GGCGACACCGTGGTGGTGCATAGC/ CGGTAAGCCCCGCGTTGCTTGGC	257	S1
	Putative regulator *tnpM*	tnpM-F/R	TGGAAACACCCGACTGCCTTGCG/ CGGCGGCGAATCACAAGCGTCCG	318	S3
	Mercuric ion reductase *merA*	merA-F2/R	AAGGCGCACGTGTCACGCTGATC/ CCGCTGCCTTCTTCAACCACCAG	1238	S1
Tn*1,2,3*	Transposase *tnpA*	Tn3-F/R	GTGCTGACTGGCAGGCAAATCGG/ GCCTGAAAATCAACCAGTCTGGC	532	S1
		RH401	CAGTGCCAGTTTGTTCC	[a]	S4
	Resolvase *tnpR*	tnpRe-F	TCAGAAGGGCACTGGTGC	[a]	This work
	R-ampicillin, $bla_{\mathrm{TEM-1}}$	bla	AACCCACTCGTGCACCCAAC	[a]	This work
		RH410	AGCGTTTCTGGGTGAGC	[a]	S4
Tn*1721*	Hypothetical protein	mpc1721-F/R	CAAATCGGCTTAGCGAACAC/ CCGGTCTGTACATTGGTATC	736	This work
		orf1-R2	TGCTCAGGTTCAGGCTTGGT	[a]	S3
	Resolvase *tnpR*	LAF/LAR	GTTCGGGTCAGCAGCTTTGAC/ GAGGGTTTCCCGGCTGATGT	510	S1
	Transposase *tnpA*	tnpA1721-F/R	AGCTCTGCCTGCTGCGCTAC/ ATGTGTTCCAGCATGTGCCG	520	S3
		tnpA1721-F2/R2	ACTGGCTGCAAAGTGTTGAA/ TCCGTAAGGGCCGAAACTTC	360	S3
	TetR repressor of *tet*(A)	tetR1721-F3/R3	TAGACGGTCTGACGACACG/ TCTCGGTCCTTCAACGTTC	572	S3
	intergenic region	IG-F	TCGTAGCAGCAGTCCTGGAC	[a]	S3
		IG-R	CGCCAAATCTCACCGTATCTCCC	[a]	S3
Tn*10*	Transposase *tnpA*	Is10-F/R2	CTACCGAGTAACACCACACCGCTC/ AGCATCAGGGCGATTAGCAGC	536	S3
		IS10	TTGAAAAGTCCTGCCTACG	[a]	S5
	TetR repressor of *tet*(B)	tetR-F/R	ACAACCCGTAAACTCGCC/ TTCCAATACGCAACCTAAAG	366	S6
		tetR-f1	GCTTAATGAGGTCGGAATCG	[a]	Malorny, unpublished
		tetR-r1	ACGCCATTGTCAGCAAATTG	[a]	Malorny, unpublished
	tetB	tetB-Fseq	CGGTGTTATTGGCCCATTAC	[a]	Malorny, unpublished
	Hypothetical protein (TetC)	tetC-F/R	ATGCCTACATCACAACAAAAACTGA/ TGCTGACAATGGCGTTTACCT	210	S3
		dtetC-f1	ACGCCATTGTCAGCAAATTG	[a]	Malorny, unpublished
		dtetC	ATAGGCATCACTTCTTGG	[a]	S5

	Hypothetical protein (TetD)	tetD-F/R	ATGTGGCGAATAAAGCGGGT/ CAAACGACGAGCACGAATATAGC	108	S3
	Hypothetical protein (YbfA or GltS)	gltS	CGTTCCTATGGTCGGTGC	[a]	S5
		gltS-r4	TCCGGCATTACCATGACCAC	[a]	Malorny, unpublished
		ybfA-F	TTGCCTGCTTTGTCACCTTC	[a]	S3
	Hypothetical protein (YbeA or YdjB)	ydjB-r1	CAAGGGAGCGTAACAGATGC	[a]	Malorny, unpublished
		ydjB-f1	AGCGCTAGAGCAAGCTGGAC	[a]	Malorny, unpublished
	Hypothetical protein (YbdA or JemC)	ybdA-F	CGTACATGCCAAAGTTTCCACG	[a]	S3
IS*CR2*	*rcr2* located in IS*CR2*	ISVsa3-F/R	GGCGAAAGGTGTTCACTTTG/ GTTTCGGCTTTGTGCTTCAG	591	S1
	R-sulphonamides, *sul2*	sul2f4	CGATGGAGGCCGGTATCT	[a]	Malorny, unpublished

[a]Fragments were generated in combination with primers indicated in Table S3. An overview of PCR-mapping strategy designed to establish the structure of the integrons and transposons is shown in Figure S1. Primers used for testing the presence of resistance and virulence genes were as previously reported [S7-S9].

S1. García P, Guerra B, Bances M, Mendoza MC, Rodicio MR (2011) IncA/C plasmids mediate antimicrobial resistance linked to virulence genes in the Spanish clone of the emerging *Salmonella enterica* serotype 4,[5],12:i:-. J Antimicrobial Chemother 66: 543-549. doi: 10.1093/jac/dkq481.

S2. Mazel D, Dychinco B, Webb VA, Davies J (2000) Antibiotic resistance in the ECOR collection, integrons and identification of a novel *aad* gene. Antimicrob Agents Chemoter 44:1568-1574. doi: 10.1128/AAC.44.6.1568-1574.2000.

S3. Martínez N, Mendoza MC, Rodríguez I, Soto S, Bances M. (2007) Detailed structure of integrons and transposons carried by large conjugative plasmids responsible for multidrug resistance in diverse genomic types of *Salmonella enterica* serovar Brandenburg. J Antimicrob Chemother. 60:1227-1234. doi: 10.1093/jac/dkm336.

S4. Bailey JK, Pinyon JL, Anantham S, Hall RM. (2011) Distribution of the $\mathit{bla}_{\mathrm{TEM}}$ gene and $\mathit{bla}_{\mathrm{TEM}}$-containing transposons in commensal *Escherichia coli*. J Antimicrob Chemother. 66:745-751. doi: 10.1093/jac/dkq529.

S5. Lucarelli C, Dionisi AM, Filetici E, Owczarek, Luzzi I, et al. (2012) Nucleotide sequence of the chromosomal region conferring multidrug resistance (R-type ASSuT) in Salmonella Typhimurium and monophasic Salmonella Typhimurium strains. J Antimicrob Chemother 67:111-114. doi: 10.1093/jac/dkr391.

S6. Morsczeck C, Langendorfer D, Schierholz JM. (2004) A quantitative real-time PCR assay for the detection of *tetR* of Tn*10* in *Escherichia coli* using SYBR Green and the Opticon. J Biochem Biophys Methods 59:217-227

S7. Beutlich J, Jahn S, Malorny B, Hauser E, Hühn S, et al. (2011) Antimicrobial resistance and virulence determinants in European *Salmonella* Genomic Island 1-positive *Salmonella enterica* isolates from different origins. Appl Environ Microbiol 77: 5655-5664. doi: 10.1128/AEM.00425-11.

S8. Hopkins KL, Wootton L, Day MR, Threlfall EJ. (2007) Plasmid-mediated quinolone resistance determinant *qnrS1* found in *Salmonella enterica* strains isolated in the UK. J Antimicrob Chemother 59:1071-1075.

S9. Rodríguez I, Guerra B, Mendoza MC, Rodicio MR. (2011) pUO-SeVR1 is an emergent virulence–resistance complex plasmid of *Salmonella enterica* serovar Enteritidis. J Antimicrob Chemother 66:218-220. doi: 10.1093/jac/dkq386.
