# Supplementary material for: Diversity of Plasmids Encoding Virulence and Resistance Functions in Salmonella enterica subsp. enterica Serovar Typhimurium Monophasic Variant 4,[5],12:i:- Strains Circulating in Europe
Source: PLoS One. 2014 Feb 26;9(2):e89635. doi: 10.1371/journal.pone.0089635 (PMC3935914; doi:10.1371/journal.pone.0089635)
Supplement: Table S3 — Overlapping PCRs designed to characterize integrons, transposons and IS CR elements. (PDF) [file pone.0089635.s004.pdf]

Table S3. Overlapping PCRs designed to characterize integrons, transposons and IS*CR* elements.

PCR number	Fragment	Primer names[a]	Expected Amplicon size (pb)	Expected Genes			Results pMVN-STm RV1	RV2	RV3	RV4	RV5	RV6	VR1	VR2
**Integrons**														
[1]	*intI1-qacEΔ1*	intI1-R2/qacEΔ1-R	650	*intI1*	i[b](empty)	*qacEΔ1*	+	+	–	–	–	–	–	–
[2]	*qacEΔ1-sul1*	qacEΔ1-F/sul1-R	798	*qacEΔ1*	*sul1*		+	+	–	–	–	–	–	–
[3]	i[c]-*sul1*	5'CS/sul1-R	1027	i[b](empty)	*qacEΔ1*	*sul1*	+	+	–	–	–	–	–	–
[4]	*intI1-dfrA12*	intI1-R2/dfrA12-R	767	*intI1*	*dfrA12*		–	–	+	+	+	+	+	+
[5]	*dfrA12-aadA2*	dfrA12-F/aadA2-R	1672	*dfrA12*	*orfF*	*aadA2*	–	–	+	+	+	+	+	+
[6]	5'CS-*aadA2*	5'CS/aadA2-R	1780	*dfrA12*	*orfF*	*aadA2*	–	–	+	+	+	+	+	+
			2501	*estX*	*psp*	*aadA2*	–	+	–	–	–	–	–	–
[7]	*dfrA12*-3'CS	dfrA12-F/3'CS	1800	*dfrA12*	*orfF*	*aadA2*	–	–	–	–	–	–	–	–
[8]	*aadA2-qacEΔ1*	aadA2-F/qacEΔ1-R	1100	*aadA2*	*qacEΔ1*		–	–	–	–	–	–	–	–
[9]	*aadA2-sul1*	aadA2-F/ sul1-R	1650	*aadA2*	*qacEΔ1*	*sul1*	–	–	–	–	–	–	–	–
[10]	*orfF-cmlA*	orf-F/cmlA-R	2096	*orfF*	*aadA2*	*cmlA1*	–	–	+	+	+	+	+	+
[11]	*orfF-qacH*	orf-F/qacH-R	1463	*orfF*	*aadA2/1*	*qacH*	–	–	–	–	–	–	–	–
[12]	*aadA2-qacH*	aadA2-F/qacH-R	1142	*aadA2/1*	*qacH*		–	–	–	–	–	–	–	–
[13]	*cmlA1-qacH*	cmlA-F/qacH-R	2229	*cmlA1*	*aadA1*	*qacH*	–	+	+	+	+	+	+	+
[14]	*intI1-estX*	intI1-R2/estX-R	1137	*intI1*	*estX*		–	+	–	–	–	–	–	–
[15]	*estX-psp*	estX-F/psp-R	1143	*estX*	*psp*		–	+	–	–	–	–	–	–
[16]	*psp-aadA2*	psp-F/aadA2-R	1433	*psp*	*aadA2*		–	+	–	–	–	–	–	–
[17]	*psp-cmlA*	psp-F/cmlA-R	2584	*psp*	*aadA2*	*cmlA1*	–	+	–	–	–	–	–	–
[18]	*qacH-sul3*	qacH-F/sul3	1565	*qacH*	*tnpAIS440*	*sul3*	nd[c]	+	+	+	+	+	+	+

**Transposons and IS*CR* elements**															
[19]	*tnpR-bla*	tnpR-R1/bla	406	*tnpR*	$bla_{\text{TEM-1}}$			+	+	nd	nd	nd	nd	+	+
[20]	*tnpA-bla*	RH401/RH410	1543	*tnpA*	*tnpR*	$bla_{\text{TEM-1}}$		+[d]	+[d]	nd	nd	nd	nd	+[d]	+[d]
[21]	IS*10-ybfA*	Is10-F/ybfA-F	1375	*IS*10	*ybfA*			nd	nd	–	–	–	–	nd	nd
[22]	IS*10-ybfA*	IS10/ybfA-F	931	*IS*10	*ybfA*			nd	nd	–	–	–	–	nd	nd
[23]	IS*10-ybfA*	IS10/gltS	736	*IS*10	*ybfA*			nd	nd	+	+	+	+	nd	nd
[24]	*ybfA-ybeA*	gltS-r4/ydjB-r1	1554	*ybfA*	*ybeB*	*ybdA*		nd	nd	+	+	+	+	nd	nd
[25]	*ybeA-tetR*	ydjB-f1/tetR-f1	1470	*ybeA*	*ybdA*	*tetR*		nd	nd	+	+	+	+	nd	nd
[26]	*ybdA-tetR*	ybdA-F/tetR-F	908	*ybdA*	*tetR*			nd	nd	+	+	+	+	nd	nd
[27]	*tetR-tetC*	tetR-r1/dtetC-f1	1650	*tetR*	*tet*(B)	*tetC*		nd	nd	+	+	+	+	nd	nd
[28]	*tetC-IS*10	dtetC/IS10	1178	*tetC*	*tetD*	*IS*10		nd	nd	–	–	–	–	nd	nd
[29]	*tetB-IS*10	tetBF-seq/IS10	1709	*tet*(B)	*tetC*	*tetD*	IS*10*	nd	nd	–	–	–	–	nd	nd
[30]	*orf1-tnpR*	orf1-R2/tnpR1721-R3	1208	*mcp*	*tnpR*			–	–	nd	nd	nd	nd	nd	nd
[31]	*tnpR-tnpA*	LAF/tnpA1721-R	1220	*tnpR*	*tnpA*			+	+	nd	nd	nd	nd	nd	nd
[32]	*tnpA-tetR*	tnpA1721-F2/tetR1721-F3	1294	*tnpA*	*tetR*			+	+	nd	nd	nd	nd	nd	nd
[33]	*tetR-tet*(A)	tetR1721-R3/tetA-R	1814	*tetR*	*tet*(A)			+	+	nd	nd	nd	nd	nd	nd
[34]	*tnpA-tet*(A)	tnpA1421-F2/tetA-R	2541	*tnpA*	*tetR*	*tet*(A)		+	+	nd	nd	nd	nd	nd	nd
[35]	*tet*(A)-IG	tet(A)-F/IG-R	1726	*tetA*	IG			+	+	nd	nd	nd	nd	nd	nd
[36]	IG-*tnpA*	IG-F/tnpA1721-R2	2078	IG	*tnpA*			+	+	nd	nd	nd	nd	nd	nd
[37]	*rcr2-sul2*	ISVsa3-F/sul2f4	1468	IS*CR2*	*sul2*			+	+	nd	nd	nd	nd	nd	nd

[a]Primer sequences are compiled in Table S2 except for sul3 (5'-3'): ATTCTTGATCCGGGTATGGG. An overview of PCR-mapping strategy designed to establish the structure of the integrons and transposons is shown in Figure S1.

[b]i, integron variable region.

[c]nd, PCR not carried out for the indicated plasmids since they were confirmed as negative by simplex-PCR for the two genes involved in the overlapping reaction.

[d]The yielded amplicon was sequenced. The fragment size shown corresponds to Tn*2* transposon.
